# Surface guided frameless positioning for lung stereotactic body radiation therapy

**DOI:** 10.1002/acm2.13370

**Published:** 2021-08-18

**Authors:** Sebastian Sarudis, Anna Karlsson, Anna Bäck

**Affiliations:** ^1^ Department of Radiation Physics Sahlgrenska Academy University of Gothenburg Göteborg Sweden; ^2^ Department of Medical Physics County Hospital Ryhov Jönköping Sweden; ^3^ Department of Therapeutic Radiation Physics Sahlgrenska University Hospital Göteborg Sweden

**Keywords:** immobilization, motion management, SBRT, SGRT

## Abstract

**Background and purpose:**

When treating lung tumors with stereotactic body radiation therapy (SBRT), patient immobilization is of outmost importance. In this study, the intra‐fractional shifts of the *patient* (based on bony anatomy) and the *tumor* (based on the visible target volume) are quantified, and the associated impact on the delivered dose is estimated for a frameless immobilization approach in combination with surface guided radiation therapy (SGRT) monitoring.

**Methods:**

Cone beam computed tomographies (CBCT) were collected in free breathing prior and after each treatment for 25 patients with lung tumors, in total 137 fractions. The CBCT collected after each treatment was registered to the CBCT collected before each treatment with focus on bony anatomy to determine the shift of the *patient*, and with focus on the visible target volume to determine the shift of the *tumor*. Rigid registrations with 6 degrees of freedom were used. The patients were positioned in frameless immobilizations with their position and respiration continuously monitored by a commercial SGRT system. The patients were breathing freely within a preset gating window during treatment delivery. The beam was automatically interrupted if isocenter shifts >4 mm or breathing amplitudes outside the gating window were detected by the SGRT system. The time between the acquisition of the CBCTs was registered for each fraction to examine correlations between treatment time and *patient* shift. The impact of the observed shifts on the dose to organs at risk (OAR) and the gross tumor volume (GTV) was assessed.

**Results:**

The shift of the *patient* in the CBCTs was ≤2 mm for 132/137 fractions in the vertical (vrt) and lateral (lat) directions, and 134/137 fractions in the longitudinal (lng) direction and ≤4 mm in 134/137 (vrt) and 137/137 (lat, lng) of the fractions. The shift of the *tumor* was ≤2 mm in 116/137 (vrt), 123/137 (lat) and 115/137 (lng) fractions and ≤4 mm in 136/137 (vrt), 137/137 (lat), and 135/137 (lng) fractions. The maximal observed shift in the evaluated CBCT data was 4.6 mm for the *patient* and 7.2 mm for the *tumor*. Rotations were ≤3.3ᵒ for all fractions and the mean/standard deviation were 0.2/1.0ᵒ (roll), 0.1/0.8ᵒ (yaw), and 0.3/1.0ᵒ (pitch). The SGRT system interrupted the beam due to intra‐fractional isocenter shifts >4 mm for 21% of the fractions, but the patients always returned within tolerance without the need of repositioning. The maximal observed isocenter shift by the SGRT system during the beam holds was 8 mm. For the respiration monitoring, the beam was interrupted at least one time for 54% of the fractions.

The visual tumor was within the planned internal target volume (ITV) for 136/137 fractions in the evaluated CBCT data collected at the end of each fraction. For the fraction where the tumor was outside the ITV, the D_98%_ for the GTV decreased with 0.4 Gy. For the OARs, the difference between planned and estimated dose from the CBCT data (D_2%_ or D_mean_) was ≤2.6% of the prescribed PTV dose. No correlation was found between treatment time and the magnitude of the *patient* shift.

**Conclusions:**

Using SGRT for motion management and respiration monitoring in combination with a frameless immobilization is a feasible approach for lung SBRT.

## 1 INTRODUCTION

Stereotactic body radiation therapy (SBRT) is a treatment method that is used for treating inoperable early stage non‐small cell lung cancer (NSCLC) or oligometastatic disease with metastases in the lung. In contrast with conventional radiation therapy, the treatment dose is given in fewer (1–10) fractions with inhomogeneous dose distributions (variation from 100% up to 160% of the prescribed absorbed dose for the target volume) and high absorbed doses per fraction (7–18 Gy).^1^ This approach has been reported to improve local tumor control for both NSCLC and oligometastatic disease compared to conventional radiation therapy, with local control rates of approximately 90%.^1^, ^2^, ^3^, ^4^, ^5^, ^6^ Due to the high dose per fraction, the tumors treated with SBRT should preferably be small (diameter ≤5 cm) and the treatment margins used to ensure adequate dose coverage during the treatment delivery should be kept to a minimum.^7^ The patient set‐up at each treatment fraction and control of the intra‐fractional patient shift and motion during treatment delivery is therefore crucial.

Immobilization devices, such as the stereotactic body frame (SBF) (Elekta AB, Uppsala, Sweden)^8^ or the BodyFix system (Elekta AB, Uppsala, Sweden),^9^ that are used for patient set‐up and control of the intra‐fractional shifts and motion during SBRT in the lungs are often complicated to use and uncomfortable for the patients. In recent years, surface guided radiation therapy (SGRT) has become an option for patient set‐up and monitoring patient motion during treatment delivery. SGRT is also capable to track the chest wall motion of the patient, which can act as a surrogate for respiratory motions and can thereby be used to trigger breathing adapted treatments. SGRT is a non‐invasive technique that uses light of different wavelengths to determine the position, posture, and respiration amplitude of a patient.^10^ Using SGRT for patient set‐up, motion management, and respiration monitoring could replace earlier mentioned immobilization devices and make the treatment procedure more tolerable for the patients. In order to assess appropriate treatment margins for SBRT of the lung when using SGRT, the accuracy and the potential to detect intra‐fractional shifts with the current SGRT‐system must be known. Catalyst HD^TM^ (c‐rad AB, Uppsala, Sweden) is a commercially available SGRT system that has been shown to have high positioning accuracy for both phantoms and patients.^11^, ^12^, ^13^ The system can be used to monitor the patient position, motion, and respiration during the delivery of a radiation treatment and set to interrupt the radiation beam if the patient breathes or moves outside preset tolerances.

In this study, the intra‐fractional *patient* motion during treatment delivery and the intra‐fractional *patient* and *tumor* shift between the end and the start of the treatment fractions were studied for a frameless immobilization used in combination with SGRT for motion management and respiration monitoring. The purpose was to quantify the magnitude of the occurring shifts and motions and to estimate the associated impact on the delivered dose in order to determine if this is a feasible immobilization approach for SBRT in the lung.

## 2 MATERIALS AND METHODS

Data from 25 consecutive patients with lung tumors that were treated with SBRT were included in the study under the general ethical approbation by the Swedish Ethical Review Authority dnr 2014/296‐31. The diameter of the tumor volume for the included patients was restricted to a maximal value of ≤5 cm. Tumors with larger diameter than 5 cm are not treated with SBRT at our department.

All the patients were positioned in a half‐body‐sized vacuum cushion placed on a Wingstep (Elekta AB, Stockholm Sweden) with their legs resting on a Prostep (Elekta AB, Stockholm Sweden). Prior to treatment, all patients performed a conventional computed tomography (CT) scan as well as a 4‐dimensional computed tomography (4DCT) scan in free breathing on a Toshiba Aquilion Large Bore CT (Canon Medical Systems, Tustin CA, USA). The respiratory signal for the 4D‐reconstruction was acquired using the Senintel^TM^ system (c‐Rad AB, Uppsala, Sweden) with the gating point placed approximately 5 cm inferior of the processus xiphoideus. Ten equidistant time phases were used to reconstruct the 4DCT. The conventional CT scan was used for delineation of organs at risk (OAR) and treatment planning. The 4DCT scan was used for the definition of the target volumes (gross tumor volume [GTV], internal target volume [ITV], and planning target volume [PTV]). It was also used to determine the gating window width that was later used during treatment delivery to assure that the patient did not breathe with higher or lower amplitudes than during the collection of the 4DCT and to determine the range of motion of the tumor.

The GTV, including breathing motions, was delineated as the tumor tissue seen with lung window settings in the maximum intensity projection (MIP) of the reconstructed 4DCT phases and the ITV was set equal to the GTV. The PTV was created using a 5 mm symmetrical margin around the ITV.

In accordance with the local clinical guidelines, the acquired 4DCT of each patient was examined by an oncologist and a physicist directly after acquisition to determine the tumor motion. If the unrestricted range of the tumor motion is within 15 mm, the patient will be treated in free breathing (FB) during the full breathing cycle. If the motion is larger the size of the high dose region and its vicinity to organs at risk will be evaluated. This evaluation can lead to a clinical decision to treat the patient in FB during the full breathing cycle even though the tumor motion was larger than 15 mm. If treating in FB has been deemed inappropriate, the guidelines recommend delivering the treatment either in breath hold (BH) or, if not possible, during end‐exhalation using only selected phases of the breathing cycle to minimize target motion. This was not necessary for any of the included patients, since their unrestricted tumor motions were either small (≤ 15 mm) (all patients except pat#13, Table 1) or the resulting volume of the ITV was small (8.9 cm^3^) (pat#13, Table 1), making the planning target volume (PTV) acceptable (37.0 cm^3^).

**TABLE 1:** Fractionation, treatment technique, tumor characteristics (location, motion vector and volume) and patient characteristics (age and body mass index [BMI]) for the patients included in this study

Patient #	Fractionation	Technique	Tumor characteristics			Patient characteristics	
			Location	Motion vector [mm]	Volume [cm3]	Age [yr]	BMI
1	15 Gy x 3	CA	Upper lobe (R)	3	15.5	83	20.6
2		CA	Middle lobe (R)	13	16.3	67	31.6
3		CA	Lower lobe (R)	12	20.2	63	22.9
4		CA	Lower lobe (R)	6	7.6	89	21.1
5		CA	Middle lobe (R)	10	10.3	66	37.2
6	12 Gy x 4	CA	Lower lobe (R)	13	23.3	85	26.8
7		CA	Lower lobe (R)	9	26.4	75	26.7
8		CA	Lower lobe (L)	11	17.1	81	23.2
9		CA	Upper lobe (R)	2	3.4	75	19
10		VMAT	Upper lobe (L)	10	40.7	87	28
11		VMAT	Lower lobe (R)	15	49.9	83	20.3
12		VMAT	Lower lobe (R)	8	11.3	87	25.7
13		CA	Lower lobe (R)	35	8.9	73	26.8
14		VMAT	Lower lobe (R)	15	23.9	79	31.6
15	6 Gy x 6	VMAT	Middle lobe (R)	3	52.6	76	26.8
16	7 Gy x 8	VMAT	Middle lobe (R)	2	5.0	59	22.9
17		VMAT	Upper lobe (L)	3	8.1	89	24.2
18		CA	Lower lobe (R)	14	7.6	94	29.7
19		VMAT	Lower lobe (R)	15	11.2	81	18.8
20		VMAT	Lower lobe (R)	12	47.7	73	24.8
21		VMAT	Lower lobe (L)	7	1.3	76	25.4
22		VMAT	Upper lobe (R)	1	1.9	78	23.5
23		VMAT	Lower lobe (L)	5	6.2	66	38.2
24		VMAT	Lower lobe (L)	6	24.6	69	30.6
25		VMAT	Lower lobe (L)	7	23.9	75	21.1

Note: CA = conformal arc, (R) = Right lung, (L) = Left lung, VMAT = volumetric modulated arc therapy.

All patients in this study were thus treated in FB over the full breathing cycle. To mitigate the risk of large inter‐fractional and intra‐fractional breathing differences, an exception gating technique was used during treatment delivery using the SGRT system to monitor the breathing amplitude of each patient. The exception gating window width used during treatment delivery was set to encompass the normal breathing amplitude recorded during the 4DCT with an additional margin of 1 mm in each direction. In this way, if the patient would breathe with higher or lower amplitudes than during the 4DCT, the treatment delivery would be automatically stopped by the SGRT system. The range of the unrestricted tumor's center of mass motion for the included patients was 1–31 mm (mean 8.7 mm) in the longitudinal (inferior‐superior) direction, 0–15 mm (mean 2.6 mm) in the vertical (anterior‐posterior) direction and 0–8 mm (mean 1.7 mm) in the lateral (left‐right) direction, which is within previously reported ranges of lung tumor motions.^14^, ^15^

The prescribed absorbed dose to the PTV and number of treatment fractions were dependent on the location of the tumor within the lung with 15 Gy × 3 for peripherally located tumors, 12 Gy x 4 for tumors with broad chest wall contact, and 6 Gy x 6 or 7 Gy x 8 for centrally located tumors. In total, 137 fractions were included and evaluated. The treatments were planned in the Eclipse treatment planning system version 13.6 (Varian Medical Systems, Palo Alto, USA) using either a conformal arc (CA) or volumetric modulated arc therapy (VMAT) technique with five half arcs. Table 1 shows the fractionation, treatment technique, tumor characteristics, and patient characteristics for each patient. Inhomogeneous dose distributions were used with the aim of covering the entire PTV with the prescribed dose and as large volume as possible of the ITV with 140% of the prescribed dose. The maximal dose was restricted to <160% of the prescribed dose.

Before each treatment fraction, the patients were first positioned on the treatment couch using the Catalyst HD^TM^ system (C‐Rad AB Uppsala, Sweden). After the initial set‐up using Catalyst, orthogonal kilovoltage (kV) images were acquired and a shift based on an online image registration with focus on the bony anatomy was performed. After the first online shift had been performed, a daily cone beam CT (CBCT) was acquired during free breathing (CBCT_pre‐start_) and the final treatment position was established by shifting the treatment couch so that the blurred tumor structure in the collected CBCT_pre‐start_ was centered within the planned ITV. This CBCT, with the performed isocenter shift, was denoted as CBCT_start_. After this positioning procedure, the motion‐ and respiration management of the Catalyst system was initiated, and a reference surface of the actual patient position was captured and saved in the Catalyst software. The Catalyst system then continuously acquires optical images of the body surface and compares them to the acquired fraction specific reference surface. The software uses a combination of a rigid and a deformable registration to determine the position of the isocenter within the patient for the current treatment plan.^16^ The calculated position of the isocenter within the patient is continuously compared with the planned position of the isocenter and a deviation is presented within the software. The deformable model is weighted in such a way that surface deviations located far away from the treatment isocenter does not affect the calculation of the isocenter position within the body as much as deviations which are located closer to the isocenter. The radiation beam was automatically stopped by the Catalyst software if the patient moved during the treatment delivery so that the Catalyst calculated isocenter position in the patient deviated more than 4 mm from the isocenter position in the acquired reference position. In cases when the beam was automatically stopped, 30 sec with no action was allowed, for the patient to return within the pre‐set tolerances. If the patient did not return within the pre‐set tolerances within this time, the clinical practice was to acquire a new CBCT to reposition the patient.

The patient respiration amplitude was also monitored with the Catalyst SGRT system during the beam delivery. The patient was breathing freely within the pre‐set gating window. If the patient respiration amplitude was outside of the gating window, the beam was automatically stopped and did only resume when the respiration amplitude returned within the gating window.

In order to evaluate the intra‐fractional shifts, an additional CBCT was acquired in free breathing directly after the final treatment arc had been delivered for each fraction (CBCT_end_). The CBCT_end_ was compared to the CBCT_start_. The two CBCTs (CBCT_start_ and CBCT_end_) were registered using the automatic image registration option in Eclipse. To determine the intra‐fractional shift of the *patient*, the registrations were performed with focus on the bony anatomy within a region of interest (ROI) that was set to cover the spine of the patient. To determine the intra‐fractional shift of the *tumor*, the registrations were performed with focus on the visible target volume within the ROI which was set to cover the ITV of the actual patient +a margin of 2 cm in each direction. All registrations were performed using rigid registrations with 6 degrees of freedom (lateral, vertical, longitudinal, roll, yaw, and pitch directions). The time between the acquisition of the CBCT_pre‐start_ and the CBCT_end_ was registered for each fraction in order to determine how long the patient had been lying on the treatment couch. This information was used to examine if there was any correlation between the time the patient had been lying on the table and the observed magnitude of the intra‐fractional shifts of the *patient*. Analyses were also performed to examine if patient's age, body mass index (BMI), or tumor volume had any correlation to the magnitude of the observed intra‐fractional shifts of the *patient*. The maximal isocenter deviation during each fraction calculated by the Catalyst system, the number of beam holds due to isocenter deviations larger than tolerance, and the number of times that the patient's respiration amplitude exceeded the pre‐set gating window during beam delivery were recorded.

For each patient with at least one fraction with an observed shift of the *patient* in the CBCT registrations exceeding 2 mm, the impact on the dose to the organs at risk (OAR) listed in Table 2 was assessed. This was done by shifting the isocenter of the original treatment plan according to the registered intra‐fractional shift of the patient for each fraction and recalculating the fraction dose in the shifted position on the original 3DCT used for treatment planning. This procedure was repeated for each fraction and the shifted plans were thereafter summed in Eclipse in order to obtain the total dose to each OAR. For patients that had no fraction with observed shifts of the *patient* exceeding 2 mm, the impact on the dose to the OAR was assumed to be negligible. For the registrations focusing on the intra‐fractional shift of the *tumor*, a visual inspection was made for each of the registrations to determine if the visible tumor in the CBCT_end_ was still within the planned ITV that was automatically overlaid by Eclipse on the CBCT image. If the visible tumor was outside the planned ITV, a deformable image registration (DIR) was performed in Eclipse between the CBCT_end_ and the original treatment planning CT, and the dose was recalculated on the deformed planning CT and structures for that specific geometry and fraction. If the visible tumor was within the planned ITV, no additional absorbed dose calculations were performed to assess target coverage as the difference was assumed to be negligible. The impact on target coverage and doses to the OARs were evaluated based on dose‐volume‐histogram (DVH) parameters of interest as presented in Table 2.

**TABLE 2:** The evaluation parameters for estimating the impact of the observed intra‐fractional shifts of the *patient* and the *tumor* on the delivered dose

Target coverage	
Structure	Evaluation parameter
GTV	D_98%_

**Organs at risk**	
Structure	Evaluation parameter
Spinal cord	D_2%_
Trachea	D_2%_
Bronchial Tree	D_2%_
Esophagus	D_2%_
Heart	D_2%_
Great Vessels	D_2%_
Brachial Plexus	D_2%_
Lungs	V_20 Gy_; D_mean_
Ipsilateral lung	D_mean_
Contralateral lung	D_mean_
Ribs	D_2%_
Thoracic Wall	V_30 Gy_; V_34 Gy_; V_45 Gy_
Skin	D_2%_
Body	D_2%_

Note: The examined dose level for the thoracic wall is dependent on the number of fractions that were used for the treatment delivery (i.e. V_30 Gy_ for 3 fractions, V_34 Gy_ for 4 fractions and V_45 Gy_ for 6 or 8 fractions).

## 3 RESULTS

**FIGURE 1:**
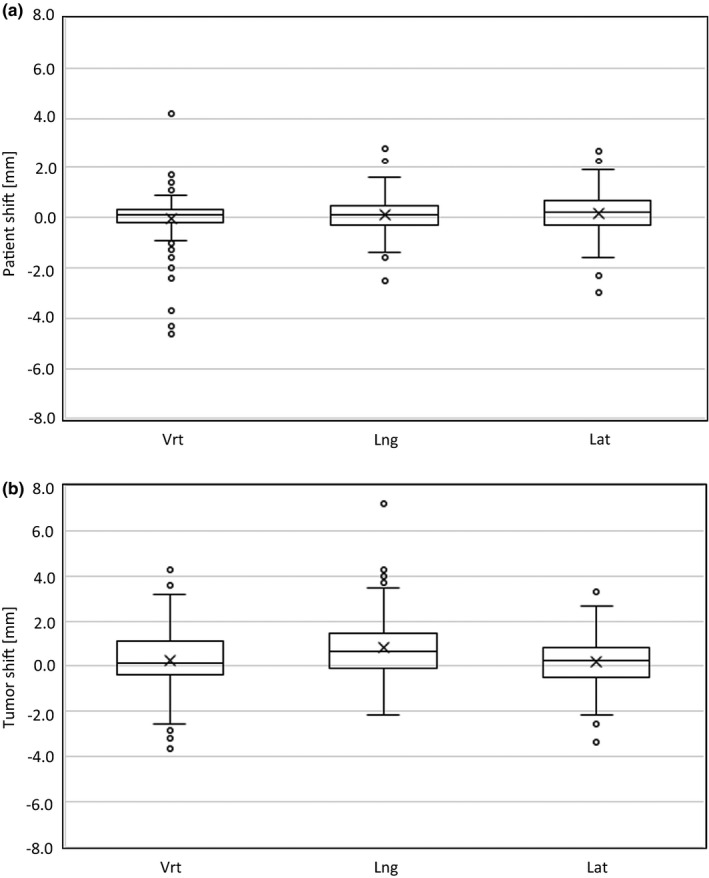
Box and Whisker plots for the intra‐fractional shifts of the *patient* (a) and the *tumor* (b) for the 137 SBRT fractions studied. The cross represents the mean value and the middle line in the box represents the median value. The box is defined by the 3rd quartile (the upper line) and the 1st quartile (the lower line). The whiskers show the extension of 1.5 x the inter‐quartile range (IQR) and the circles are outliers exceeding 1.5 x IQR

The CBCT‐measured intra‐fractional shift of the *patient* (registrations based on bony anatomy) in the translational directions (vertical [vrt], longitudinal [lng], and lateral [lat]) is presented in Figure 1a. The shift was ≤2 mm for 132/137 (96.4%) of the fractions in the vertical and lateral directions, and for 134/137 (97.8%) of the fractions in the longitudinal direction. The corresponding evaluation for the intra‐fractional shift of the *tumor* (registrations based on the visible target volume) showed ≤2 mm shift for 116/137 (84.7%) of the fractions in the vertical direction, 115/137 (83.9%) in the longitudinal direction and 123/137 (89.8%) in the lateral direction (Figure 1b). 136/137 (99.3%) of the fractions in the vertical direction, 135/137 (98.5%) in the longitudinal direction, and 137/137 (100%) of the fractions in the lateral direction had a shift of the *tumor* ≤4 mm. The corresponding values were 134/137 (97.8%) in vrt and 137/137 (100%) in lng, lat for the shifts of the *patient*. The total mean/standard deviation for the intra‐fractional shifts of the *patient* were 0.0/1.0 mm (vrt), 0.1/0.7 mm (lng), 0.2/1.0 mm (lat). For the intra‐fractional shifts of the *tumor* the corresponding values were 0.2/1.3 mm (vrt), 0.8/1.3 mm (lng), and 0.1/1.1 mm (lat).

**FIGURE 2:**
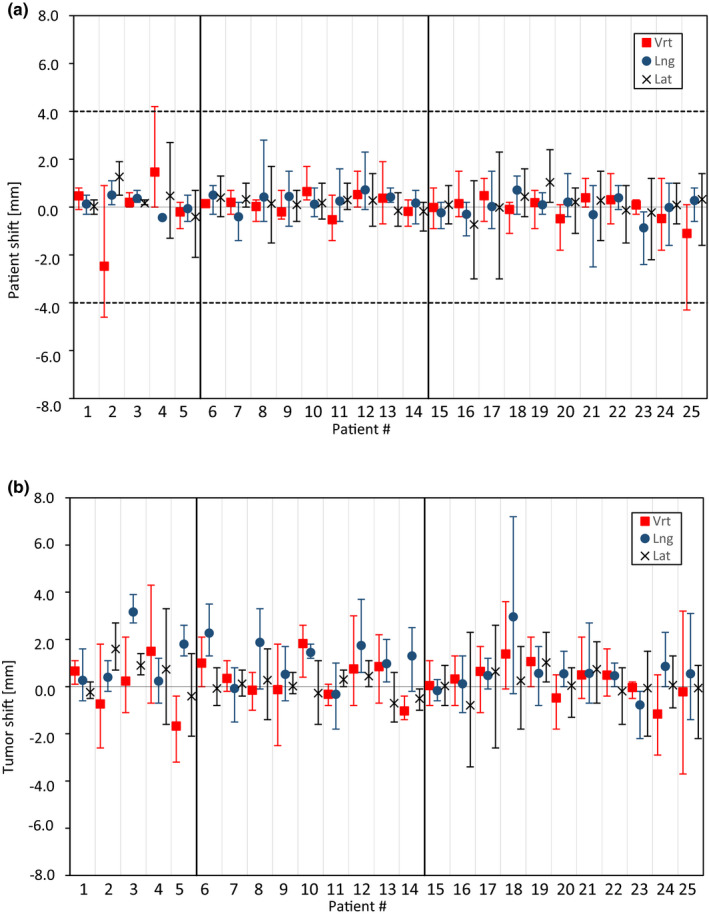
Intra‐fractional shifts of the *patients* (a) and the *tumors* (b) for each individual patient in the vertical (vrt), longitudinal (lng), and lateral (lat) directions. The points represent the mean values for all fractions and the whiskers show the maximum values. The dotted line in figure A represents the isocenter deviation tolerance used (4 mm) for motion monitoring with the surface guidance system

Figure 2a shows the CBCT‐measured intra‐fractional shifts of the *patient* for each individual patient. Three of the 137 examined fractions had an intra‐fractional shift exceeding the 4 mm isocenter deviation tolerance set for the motion monitoring in the surface guidance system (pat# 2, 4 and 25). None of the patients had an intra‐fractional shift exceeding 4.6 mm. The intra‐fractional shifts of the *tumors* are presented in Figure 2b.

**FIGURE 3:**
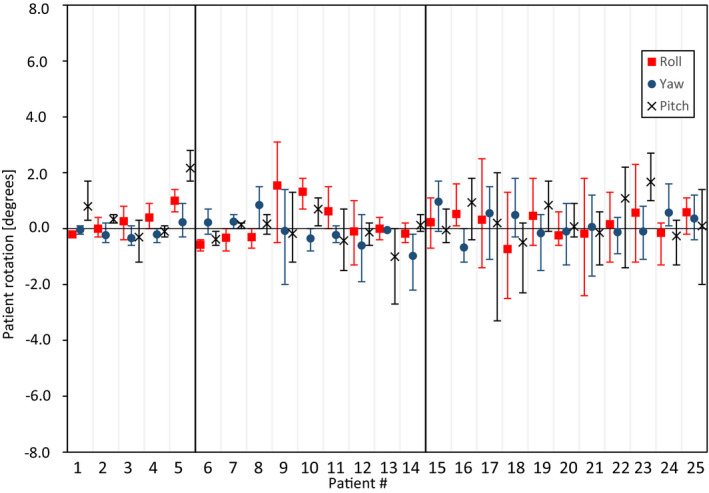
Intra‐fractional *patient* rotations for each individual patient based on CBCT registrations performed before and after each treatment fraction. The points represent the mean values for all fractions and the whiskers show the maximum values

The CBCT‐measured *patient* rotations in roll, yaw and pitch were within 3° for all fractions except one fraction for pat# 17 where the pitch rotation was 3.3ᵒ and one fraction for pat# 9 where the roll rotation was 3.1ᵒ (Figure 3). The mean/standard deviation of the *patient* rotations were 0.2/1.0ᵒ (roll), 0.1/0.8ᵒ (yaw), and 0.3/1.0ᵒ (pitch). An evaluation of the rotational directions for the intra‐fractional *tumor* motion was not performed since it was difficult to determine these for the symmetrically shaped tumors.

**FIGURE 4:**
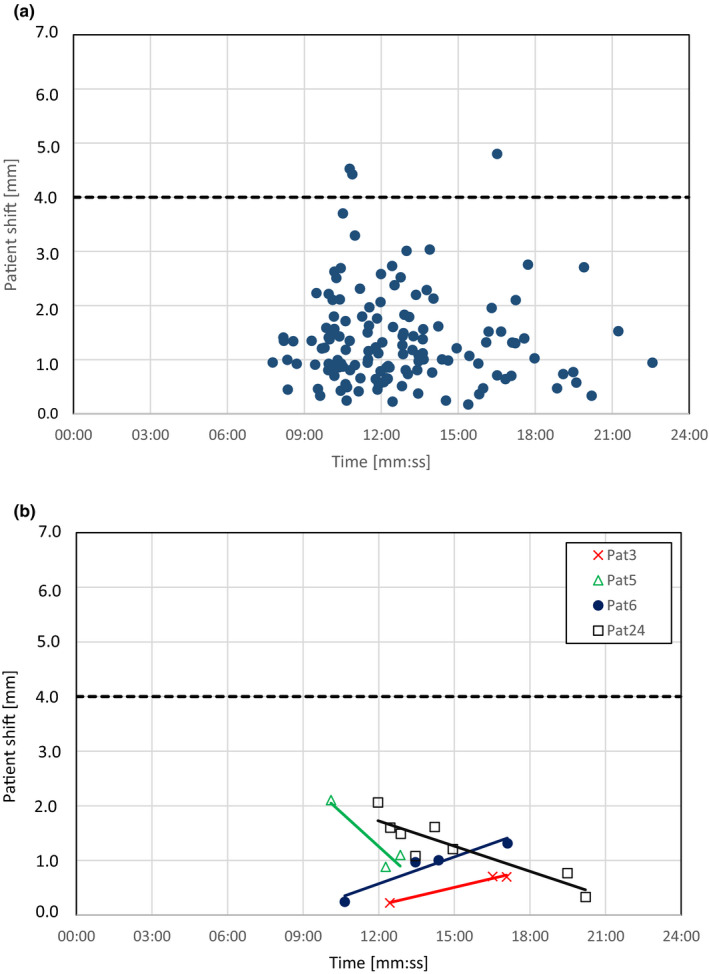
Magnitude of the observed intra‐fractional shift of the *patient* vs the registered time between CBCT_pre‐start_ and CBCT_end_ for all the 137 fractions that were evaluated (a) and for the individual patients with correlation coefficients ≥0.800 (b). A linear fit for all the patients in figure A shows a *R* ^2^‐value of 0.0023. In figure B, patient 3 and 6 have an increased patient shift with increased treatment time while patient 5 and 24 have a decreased patient shift with treatment time. The solid lines show the linear fits for the points and the dotted lines represents the isocenter tolerance used (4 mm) for patient monitoring with the surface guidance system

The treatment time, that is the time between the CBCT_pre‐start_ and CBCT_end_, for the 137 fractions that were evaluated was between 7 min 46 s and 22 min 34 s for the patients included in this study. For this time interval, no correlation was found between the treatment time and the magnitude of the intra‐fractional shift of the *patient* measured in the CBCT when looking at the whole patient group (*R* ^2^ = 0.002), Figure 4a. Evaluating each patient separately showed a positive correlation (i.e. increased shifts with increased treatment time) for two of the patients (pat# 3 and 6) (*R* ^2^ > 0.900) and a negative correlation (i.e. decreased shifts with increased treatment time) for two of the patients (pat# 5 and 24) (*R* ^2^ > 0.800), Figure 4b. For the rest of the 21 patients no correlation was found between the shift of the *patient* and the treatment time (*R* ^2^ < 0.460). Age, BMI, or tumor volume were not found to correlate with the magnitude of the intra‐fractional shift of the *patient*, *R* ^2^ = 0.012, *R* ^2^ = 0.017 and *R* ^2^ = 0.060 respectively.

None of the patients had to be repositioned during a treatment fraction. The SGRT system interrupted the beam due to intra‐fractional isocenter shifts exceeding the preset tolerance of 4 mm for 29 of the 137 treatment fractions (21%) but the patients always returned within the allowed tolerance within 30 s without the need of repositioning. In total, 72 beam holds due to intra‐fractional isocenter deviations were recorded for the included patients. The maximum isocenter deviation detected by the SGRT system during the beam holds was 8 mm (Table 3). For the respiration monitoring, the SGRT system interrupted the beam delivery because the patient's respiration amplitude extended outside the exception gating window at least one time for 54% of the fractions.

**TABLE 3:** The maximum intra‐fractional isocenter deviations recorded by the Catalyst system during treatment delivery for each patient presented together with the number of fractions where the beam was held due to these deviations and the total number of beam holds due to isocenter deviations during the entire treatment

Patient #	Max iso dev [mm]	# fractions with Beam Hold	Total # Beam Holds
1	1	0	0
2	1	0	0
3	1	0	0
4	4	1 of 3	1
5	5	1 of 3	1
6	5	3 of 4	21
7	6	2 of 4	9
8	2	0	0
9	2	0	0
10	5	1 of 4	1
11	5	2 of 4	5
12	5	1 of 4	1
13	7	3 of 4	5
14	3	0	0
15	5	1 of 6	2
16	4	1 of 8	1
17	5	1 of 8	1
18	4	1 of 8	1
19	5	3 of 8	5
20	8	5 of 8	12
21	3	0	0
22	3	0	0
23	3	0	0
24	6	3 of 8	6
25	3	0	0

Note: 11 of 25 patients had observed positional shifts of the body (i.e. *patient* shifts) exceeding 2 mm in the CBCT registrations. The impact of the intra‐fractional shift of the *patient* on the dose to the OARs for these patients is presented in Table 4.

**TABLE 4:** The difference between the estimated and the planned value for each DVH parameter for all the patients with at least one fraction with an observed shift of the *patient* in the CBCT registrations exceeding 2 mm

		Pat 2	Pat 4	Pat 5	Pat 8	Pat 12	Pat 16	Pat 17	Pat 19	Pat 21	Pat 23	Pat 25
**D_2%_** [% of prescribed PTV dose]	Spinal cord	0.3	−0.1	−0.1	0.0	0.4	−0.1	−0.2	0.5	−0.1	0.0	0.1
	Trachea	0.0	0.0	0.0	0.0	0.0	0.0	0.0	0.0	0.0	0.0	0.0
	Bronchial Tree	**2.3**	0.0	−0.5	−0.2	0.6	−0.3	−0.2	−0.7	−0.2	**−2.2**	**2.2**
	Esophagus	0.0	−0.1	−0.1	0.0	0.2	−0.2	−0.1	**2.6**	0.0	0.0	−0.1
	Heart	−0.5	0.3	0.0	0.1	0.0	−0.6	0.0	1.0	−0.1	0.0	−1.0
	Aorta	0.0	0.0	0.0	0.0	0.0	0.0	0.1	0.0	0.0	0.0	0.2
	Brachial Plexus	0.0	0.0	0.0	0.0	0.0	0.0	0.0	0.0	0.0	0.0	0.0
	Ribs	**−8.0**	0.7	0.9	−0.7	−1.3	1.3	0.0	1.1	0.8	0.0	−0.6
	Skin	0.1	−0.1	−0.2	−0.3	−0.2	−0.3	−0.1	−0.2	−0.1	−0.1	−0.3
	Body	0.3	−0.2	−0.1	−0.2	0.1	−0.1	0.0	−0.1	−0.1	−0.1	0.0
**D_mean_** [% of prescrb. PTV dose]	Lungs	0.1	−0.1	−0.1	−0.1	0.0	0.0	0.0	−0.1	0.0	0.0	0.0
	Ipsilateral lung	−0.2	0.0	−0.1	−0.1	0.0	0.0	0.0	−0.1	−0.3	0.0	0.0
	Contralateral lung	0.0	0.0	0.0	0.0	0.0	0.0	0.0	0.0	0.0	0.0	0.0
**V_20 Gy_** [%]	Lungs	0.2	0.0	−0.1	0.0	0.0	0.0	0.0	−0.1	−0.1	0.0	0.0
**V_xGy_** * [%]	Thoracic Wall	**−2.4**	0.1	0.0	0.1	−0.3	0.0	0.0	0.6	0.0	0.0	0.0

Note: The values are presented as % of the prescribed PTV dose for the D_2%_ and D_mean_ parameters, and in % of total structure volume for the V_20 Gy_ and V_xGy_ parameters. The examined dose level for the thoracic wall is dependent on the number of fractions that were used for the treatment delivery (i.e. x = 30, 34, 45 Gy for 3, 4 or 8 fractions respectively). A positive difference means that the estimated value was higher than the planned value. Differences ≥2% are presented as bold numbers.

For all cases except for the ribs for pat# 2, the difference between the planned and the delivered dose to the OAR was ≤2.6% of the prescribed PTV dose. For the volume constraints, V_20 Gy_ to the lungs and V_XGy_ to the thoracic wall, the difference was ≤2.4%. For 1 of 137 fractions the visible tumor in the CBCT_end_ was outside the planned ITV. This occurred in one of the four fractions for patient #13. The effect of this shift on the dose coverage to the GTV in that specific fraction was a decrease of the D_98%_ with 0.4 Gy, from 16.2 Gy to 15.8 Gy. In patient #11, a systematic miss alignment of 3 mm in the vertical direction was observed for the patient position in the CBCT_start_ data for three of the four treatment fractions. This resulted in the visible tumor being outside the planned ITV for these fractions. If the systematic error was corrected for, the visible tumor was within the planned ITV for all the fractions and was therefore considered to be a patient with no tumor shift larger than the ITV‐margins in this study.

## 4 DISCUSSION

The demonstrated results from this study show that using a relatively simple immobilization with Wingstep, vacuum cushion, and Prostep together with a SGRT system to monitor patient motion and respiration during treatment delivery is feasible for delivering high accuracy SBRT in the lung. The observed translational shifts of the patients in this study are comparable to the accuracy presented in previous studies for more complex immobilizations such as the Stereotactic Body Frame (SBF) (Elekta AB, Uppsala, Sweden) with reported shifts ≤4.7 mm^17^ or the dual vacuum BodyFix system (Elekta AB, Uppsala, Sweden) with reported shifts ≤3.2 mm.^18^ The same applies to the observed rotations which in this study were ≤3.3ᵒ, compared to the maximal reported rotation of 3.0ᵒ for the BodyFix^18^ and 1.4ᵒ for the SBF.^17^ This study furthermore provides information on how the patients move and breathe during the treatment delivery as the patient is monitored with the SGRT system. Monitoring the patient with a SGRT system reduces the risk of irradiating the patient in a different geometry than what was planned for. The information from the SGRT system showed that beam interruptions due to temporary deviations in patient position or respiration are not uncommon (72 beam holds due to intra‐fractional isocenter deviations >4 mm and beam holds at least one time in 54% of the fractions due to deviations in respiration). In another recent study by Heinzerling et al.^19^ where they evaluated SGRT in combination with image guidance for intra‐fraction motion monitoring during SBRT treatments of the lung and abdomen, they reported beam holds in 10% of the 335 fractions that were studied. A beam hold was observed for 25/71 patients. The threshold for automatic beam interruptions in their study was defined as a 2 mm translation along any axis with a maximum duration of 2 s out of tolerance. However, Heinzerling et al. used a different SGRT system than the one used in our study, and it is not clear if the estimated deviations are comparable to the isocenter deviations calculated by the SGRT system used in our study. These type of data are not commonly reported in the literature for SBRT immobilization approaches and further clinical data on intra‐fractional patient shifts during radiotherapy delivery are valuable in order to provide information on the stability of the position and breathing of the patient. Moreover, we present estimations of the delivered dose to the target and to organs at risk for the observed patient shifts. Those estimations add support to the statement that high accuracy immobilization is achievable without the need of the more complex and complicated traditional SBRT immobilizations.

The magnitude of lung tumor motions in FB are more commonly reported, for example, Sarudis et al.^14^ and Keall et al.^15^ In our study, the ITV concept was used for treatment planning with the purpose to encompass the position of the tumor during the entire treatment. When comparing the CBCT registrations before and after each fraction, this was the case for 136/137 fractions. Two different studies on SBRT treatments using more complex immobilizations reported shifts of lung tumors that were estimated using a similar method to ours, that is, by comparing repeated CT or CBCT‐scans to either the initial CT used for treatment planning^17^ or to a CBCT collected before every treatment fraction.^20^ In those studies, the maximal observed intra‐fractional internal *tumor* shift for patients treated in the Elekta Stereotactic Body Frame was found to be 10 mm^17^ and the mean intra‐fractional variation in target position reported for three different immobilization devices (SBF, BodyFIX and Alpha Cradle (KGF Enterprises, Chesterfield, MI)) was 2.3 mm with a standard variation of 2.1 mm and a maximal deviation of 17.5 mm.^20^ In our study, we found mean, standard deviation, and maximal observed intra‐fractional *tumor* shifts of 0.8, 1.3, and 7.2 mm, respectively, which are superior to the results from the earlier studies. One factor that might have an influence on the different results in the studies is the motion distribution of the tumors of the included patients. The mean 3D vector of the tumor motion for the included patients in our study was 9.2 mm with a range of 1–35.4 mm. The corresponding values are not reported in the earlier studies. Furthermore, none of these studies, including our own, measure the actual *intra*‐*fractional* motion since no images were acquired during the treatment delivery.

The observed shifts found for the *tumors* were larger than the shifts of the *patients*. This is expected since the patient is lying relatively still on the treatment table while the tumor inside the patient moves due to the patient's respiration, and no measures, such as abdominal compression plates, were used to restrict the respiration and thus tumor motion. The estimation of the *tumor* shifts also include larger uncertainties compared to the estimation of the *patient* shifts. The CBCTs before and after the treatments are acquired in free breathing (FB‐CBCT), which means that the tumor does not have distinct edges in the CBCT images since the tumor is moving during image acquisition. This causes a blurry image which makes it more difficult to determine the exact position of the tumor in those images. The accuracy could have been improved by acquiring 4D‐CBCT scans, but that was not technically available in our department. However, the FB‐CBCTs still provide information on where the tumor is positioned during the vast majority of its motion trajectory, if the tumor remains inside the ITV and if the position of the blurry tumor has changed between the CBCT collected at the start of each fraction (CBCT_start_) and the CBCT collected at the end of each fraction (CBCT_end_). Furthermore, if an extreme position of the tumor is not captured during the CBCT acquisition, the time the tumor spends in that position is relatively short and will have a smaller influence on the delivered dose to the tumor. Due to the longer time that is required to acquire a FB‐CBCT, it resembles an average intensity projection (AIP) reconstruction of a 4DCT or a slow CT.^21^

The time between the collection of the CBCT_pre‐start_ and the CBCT_end_ was between 7 min 46 s and 22 min 34 s for the patients included in this study. When looking at the whole patient group, no correlation was found between the treatment time and the magnitude of the shift of the *patient*. Evaluating each patient separately showed a positive correlation for two of the patients and a negative correlation for two other patients. However, three of these patients were treated with only four fractions or less, which makes the statistical evaluation uncertain. The tendency for moving is probably individual and depends on each patient, since no correlation could be found between observed intra‐fractional shifts of the patient and patient characteristics (age, BMI) or tumor volume. If a patient is comfortable in the current immobilization device, the likelihood of remaining immobilized for a longer period of time is presumably larger. Previous studies have shown contradictory results on this topic. Purdie et al.^22^ and Shah et al.^20^ demonstrated a correlation between treatment time and the magnitude of patient motion when analyzing composite groups of patients. However, the evaluated time interval in both of these studies were larger than in our study (range 15–60 min in the study by Purdie et al. and time intervals of 15.8–24.8 min or 15.9–27.1 min for two different patient groups in the study by Shah et al.^20^ On the other hand, studies like Sonke et al.^23^ and Li et al.^24^ did not find such correlations for their studied patient groups even though the time intervals that were studied were in the range of 6–60 min.

The impact of a *patient* shift on the dose to an OAR is dependent on the direction of the shift and the proximity of the OAR to the PTV, that is, the high dose region. If the shift moves the OAR toward, or into, the high dose region and the distance between the OAR and PTV is small, then the impact on the dose to the OAR is larger than in the opposite scenario. This was seen for the ribs of pat# 2, where a mean *patient* shift of 3.6 mm resulted in a dose difference of 8% of the prescribed PTV dose, compared to for example pat# 4, where a mean *patient* shift of 2.9 mm only resulted in a dose difference to the ribs of 0.7% of the prescribed PTV dose. The ribs of pat# 2 where located much closer to the PTV than the ribs of pat# 4. If the shift is non‐systematic, the sum of the dose contributions to an OAR from all fractions can smooth out such an impact. Regarding target coverage, except for one fraction, the visible tumor was within the ITV which means that target coverage is ensured.^25^, ^26^ For the case where the visible tumor in the CBCT_end_ was outside of the ITV, the re‐calculated dose for the maximal observed deviation showed a difference in the D_98%_ to the GTV of −0.4 Gy for that fraction.

The SGRT system was monitoring the breathing amplitude and the isocenter position by observing the patient surface, not the actual tumor. Since no live fluoroscopy images or tracking of fiducial markers have been used, we cannot conclude on the correlation between the surface monitoring and the actual tumor position during treatment. However, previous studies have demonstrated that a high correlation between the respiratory waveform collected by tracking the patient surface and the internal 3D tumor motion in the inferior‐superior direction is possible to obtain if the region of interest (ROI) is placed on the central parts of the patient where the surface has a high amplitude and the position of the ROI can be maintained between and during fractions.^27^, ^28^, ^29^, ^30^, ^31^ The use of SGRT for respiration monitoring using exception gating is therefore a quality improvement as it stops the beam if the breathing amplitude of the patient drastically changes from what was recorded during the collection of the 4DCT used for target delineation.

## 5 CONCLUSIONS

Using a relatively simple immobilization with Wingstep, vacuum cushion and Prostep together with SGRT for motion management and respiration monitoring was found to be a feasible approach for lung SBRT. Comparing the position of the patient directly before and directly after each treatment fraction showed observed shifts ≤2 mm for ≥96.4% of the 137 studied fractions and the visible tumor remaining within the planned ITV for all 137 fractions, except one. The estimated delivered doses expressed in DVH‐parameters of interest for the OARs deviated ≤2.6% of the prescribed PTV dose from the planned values for all cases except for the ribs in one patient where the estimated dose decreased by 8% (3.6 Gy).

## CONFLICT OF INTEREST

The authors have no relevant conflicts of interest to disclose.

## Data Availability

The data that support the findings of this study are available on request from the corresponding author. The data are not publicly available due to privacy or ethical restrictions.
